# A Field Recombinant Strain Derived from Two Type 1 Porcine Reproductive and Respiratory Syndrome Virus (PRRSV-1) Modified Live Vaccines Shows Increased Viremia and Transmission in SPF Pigs

**DOI:** 10.3390/v11030296

**Published:** 2019-03-23

**Authors:** Julie Eclercy, Patricia Renson, Arnaud Lebret, Edouard Hirchaud, Valérie Normand, Mathieu Andraud, Frédéric Paboeuf, Yannick Blanchard, Nicolas Rose, Olivier Bourry

**Affiliations:** 1 Agence Nationale de Sécurité Sanitaire de l’Alimentation, de l’Environnement et du Travail (Anses), Unité Virologie Immunologie Porcines, BP 53, 22440 Ploufragan, France; julie.eclercy@anses.fr (J.E.); patricia.renson@anses.fr (P.R.); 2 Université Bretagne Loire, Cité internationale, 1 place Paul Ricoeur, CS 54417, 35044 Rennes, France; edouard.hirchaud@anses.fr (E.H.); mathieu.andraud@anses.fr (M.A.); frederic.paboeuf@anses.fr (F.P.); yannick.blanchard@anses.fr (Y.B.); nicolas.rose@anses.fr (N.R.); 3 Institut du Porc (IFIP), 5 Rue Lespagnol, 75020 Paris, France; 4 Porc.Spective, Groupe vétérinaire Chêne Vert Conseil, ZA de Gohélève, 56920 Noyal-Pontivy, France; a.lebret@chenevertconseil.com (A.L.); v.normand@chenevertconseil.com (V.N.); 5 Anses, Unité Génétique Virale et Biosécurité, BP 53, 22440 Ploufragan, France; 6 Anses, Unité Epidémiologie et Bien-être Porcins, BP 53, 22440 Ploufragan, France; 7 Anses, Service de Production de Porcs Assainis et Expérimentation, BP 53, 22440 Ploufragan, France

**Keywords:** PRRS virus, modified live vaccine, PRRSV-1, safety, recombination, transmission, persistence

## Abstract

In Europe, modified live vaccines (MLV) are commonly used to control porcine reproductive and respiratory syndrome virus (PRRSV) infection. However, they have been associated with safety issues such as reversion to virulence induced by mutation and/or recombination. On a French pig farm, we identified a field recombinant strain derived from two PRRSV-1 MLV (MLV1). As a result, we aimed to evaluate its clinical, virological, and transmission parameters in comparison with both parental strains. Three groups with six pigs in each were inoculated with either one of the two MLV1s or with the recombinant strain; six contact pigs were then added into each inoculated group. The animals were monitored daily for 35 days post-inoculation (dpi) for clinical symptoms; blood samples and nasal swabs were collected twice a week. PRRS viral load in inoculated pigs of recombinant group was higher in serum, nasal swabs, and tonsils in comparison with both vaccine groups. The first viremic contact pig was detected as soon as 2 dpi in the recombinant group compared to 10 and 17 dpi for vaccine groups. Estimation of transmission parameters revealed fastest transmission and longest duration of infectiousness for recombinant group. Our in vivo study showed that the field recombinant strain derived from two MLV1s demonstrated high viremia, shedding and transmission capacities.

## 1. Introduction

Porcine reproductive and respiratory syndrome (PRRS) is endemic in most pig-producing countries worldwide and is reported to be among the diseases with the highest economic impact in the modern pig industry [1,2]. The disease is mainly characterized by reproductive failure in pregnant sows, respiratory disorders, and growth retardation in piglets [3,4]. The causative agent, PRRS virus (PRRSV), belongs to the *Arteriviridae* family in the *Nidovirales* order. The viral genome consists of a positive-sense, single-stranded RNA molecule of ~15 kb coding for 10 open reading frames (ORFs) [5,6]. Genetically, PRRSV strains are divided into PRRSV-1 (mainly predominant in Europe) and PRRSV-2 (predominant in North America and Asia), sharing only 60% nucleotide sequence identity [7,8].

Since the emergence of PRRS, several modified live PRRSV-1 vaccines (MLV1s), i.e., live attenuated viral strains, have been licensed. They are currently frequently used as a tool to reduce the clinical impact of PRRSV infection and to control the within-herd dynamics of infection [9]. However, the current MLVs have been associated with certain safety concerns such as reversion to virulence [10]. Importantly, mutation and recombination events confer remarkable plasticity to the RNA genome of PRRSV, including vaccine strains [11,12,13,14,15]. Evidence of recombination between PRRSV-1 or PRRSV-2 field strains has been reported several times, especially in Asia where highly pathogenic PRRSV-2 strains circulate [16,17,18]. Regarding recombination between MLV and PRRSV field strains, most of the cases were documented for PRRSV-2 strains [19,20,21,22,23], with in some cases, increased pathogenicity of the recombinant strains compared to their parental strains [24,25]. More rarely, recombination phenomena between MLV1 strains and PRRSV-1 field strains have been described. These types of recombinant strains were identified in Great Britain by Frossard et al. [26] and also more recently in China by Chen et al. [27], who detected a recombinant strain between Unistrain^®^ PRRS and a PRRSV-1 field strain in a diseased pig. Nevertheless, no evidence of occurrence of recombination events between two MLVs (MLV1 or MLV2) has been described to date.

On a pig farm in France with documented PRRSV infection, where vaccination was successively implemented a few weeks apart, first with Unistrain^®^ PRRS and then with Porcilis^®^ PRRS vaccines, we recently identified a recombinant strain between the two commercial vaccine strains. Full-genome sequencing was performed and phylogenetic analysis displayed three recombination events (all located in ORF1 encoding the viral RNA replicase) with Unistrain^®^ PRRS vaccine as the major parent and Porcilis^®^ PRRS as the minor parent [28].

The objective of the present study was to evaluate and compare the clinical, virological, and transmission parameters of this field recombinant strain derived from two MLV1s to its two parental MLV1 strains.

## 2. Materials and Methods

### 2.1. Viruses

The recombinant strain PRRS-FR-2014-56-11-1 was isolated and propagated in pulmonary porcine alveolar macrophages (PAMs) [28,29], GenBank accession No. KY767026, corresponding to the 2nd passage. The virus titer was determined in PAMs (10^6^ TCID_50_/mL), as well as in MARC-145 (10^4.2^ TCID_50_/mL). The PAMs were obtained by bronchoalveolar lavages from lungs of specific pathogen free (SPF) piglets aged between 6 and 8 weeks old in Anses, Ploufragan-Plouzané laboratory. The MARC-145 cells were provided by Pr. Moennig from the Federal Research Centre for Virus Diseases of Animals, Tubingen, Germany.

For this animal experiment, Unistrain^®^ PRRS vaccine (HIPRA, Amer, Girona, Spain; VP-046bis strain, GenBank accession No. GU067771.1) and Porcilis^®^ PRRS vaccine (MSD, Kenilworth, NJ, USA; DV strain, GenBank accession No. KF991509.1) were used as recommended by the manufacturers in the Summary of Product Characteristics (SPC).

Equivalent titers determined in MARC-145 cells were used for inoculations: 10^4.2^ TCID_50_/mL for the recombinant strain, 10^4.8^ TCID_50_/mL for the Porcilis^®^ PRRS vaccine (batch No. A207CB01), and 10^5^ TCID_50_/mL for the Unistrain^®^ PRRS vaccine (batch No. OL502C).

### 2.2. Animal Experiment

**Figure 1:**
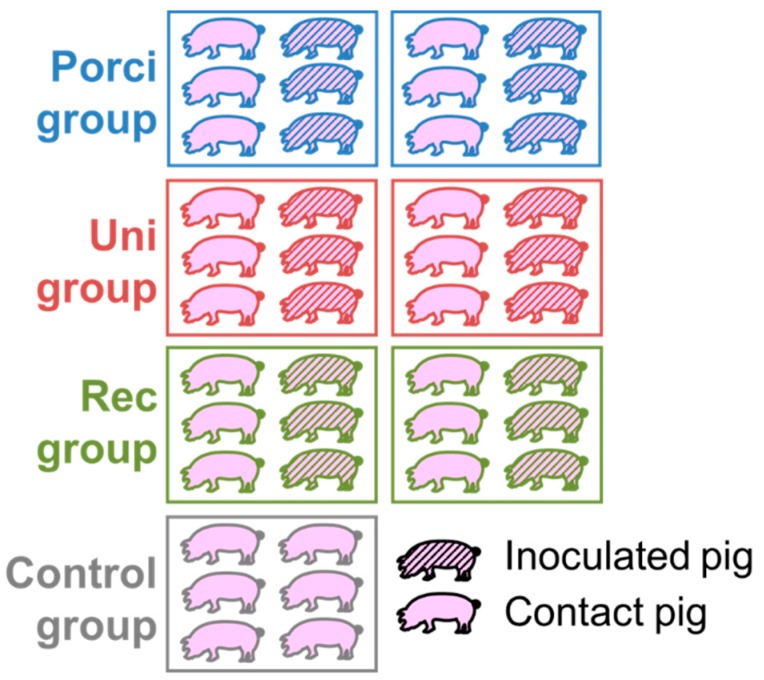
Experimental design.

The experiment was carried out in our BSL-3 animal facilities in Anses, Ploufragan-Plouzané laboratory. Forty-two seven-week-old specific pathogen free (SPF) large white piglets were randomly stratified by gender, weight, and litter and assigned to four groups housed in independent rooms, each containing two pens separated by a solid plastic partition preventing contact between pigs (Figure 1). Six piglets in each group (three per pen) were inoculated intramuscularly (IM) in the neck with 2 mL of the 2nd passage of the field recombinant strain [group: Rec] or were vaccinated, using the same procedure as the Rec group, with Porcilis^®^ PRRS [group: Porci] or with Unistrain^®^ PRRS [group: Uni], according to the manufacturers’ instructions. The control piglets (*n* = 6) were not inoculated. Twenty-four hours after inoculation, six non-inoculated contact piglets (three per pen) were added to each inoculated-group to evaluate viral transmission. Rectal temperatures, food intake, and clinical signs were monitored daily, whereas weight gain was recorded weekly, starting from 5 days pre-inoculation until the 35th day post-inoculation (dpi). Rectal temperatures over 40.0 °C were considered hyperthermia. Blood samples were taken from all pigs and nasal swabs only from inoculated and control animals before inoculation, and then twice a week until 5 weeks after inoculation, i.e., at −5, 2, 4, 7, 10, 14, 17, 21, 24, 28, 31, and 35 dpi. Sera separated from blood and nasal swabs suspended in RNA*later*™ (Qiagen, Netherlands) were stored at −80 °C until tested. All pigs were euthanized at 36–39 dpi, after anesthesia (Zoletil^®^, Virbac, Carros, France, using 15 mg/kg), followed by bleeding and then necropsied. Post-mortem examination of lesions was carried out on each pig and tissue samples were collected and stored at −80°C for further analysis.

The protocol was approved on December 13, 2016 by the Ethics Committee number 16 under reference 16-088 and was also approved by the French Ministry of Research under reference 7788-201611281003891_v1.

### 2.3. Quantification of PRRS Viral Genome Load in Sera, Nasal Swabs, and Tissue Samples

Viral RNA was extracted using the NucleoSpin RNA 8 virus kit (Macherey-Nagel, Düren, Germany) according to the manufacturer’s instructions, then PRRSV ORF-7 and porcine β-actin gene expression were quantified using an in-house duplex qRT-PCR, as previously described [30]. To be sure that the ORF7 PCR was accurate to quantify each of the 3 PRRSV strains we checked that the primers and probe were fitted for each virus and that the PCR efficiency was equivalent for each strain (93 to 96%). The genomic viral load in sera and tissue samples was quantified using a standard viral range of the recombinant strain (with a known virus titer obtained in PAMs) diluted in serum or tissue lysate collected from SPF pigs. Results were expressed as equivalent (eq) TCID_50_/mL of the sample matrix used. Virus shedding was quantified in nasal swab supernatants of inoculated and control pigs by relative quantification using the ΔΔCt method, and results were expressed in log2 R (relative amount R = 2^−ΔΔC^), as previously described [30].

### 2.4. On Farm Follow-Up of the Recombinant Strain

In 2016, 2 years after isolation of the PRRS-FR-2014-56-11-1 strain, new samples were collected from the same farm. A new PRRSV strain (PRRS-FR-2016-56-11-1) was isolated and amplified in PAMs; no propagation in MARC-145 was observed. The full-genome was sequenced at Anses Next-Generation Sequencing (NGS) platform and deposited into GenBank under accession no. MH018883. The full-genome sequence obtained was aligned with other publicly available PRRSV-1 full-genomes using the Multiple Sequence Comparison by Log-Expectation (MUSCLE) algorithm. Then, to compare both strains, the multiple alignment was submitted to the Simplot Program to screen for similarities and analyzed using the Recombination Detection Program (RDP4) software to determine homologous recombination events, as previously described [28].

### 2.5. Statistical Analyses and Transmission Parameters

Data were analyzed with R software (v.3.2.1). Rectal temperatures, daily average weight gain, and viral load in tissues were compared between the different groups for each measured point using the non-parametric Kruskal-Wallis test. Then, a Wilcoxon pairwise test with the Holm’s method for adjustment of multiple comparisons was used to compare groups with one another. The genomic viral loads in blood samples and nasal swabs for inoculated animals were compared between groups by calculating the area under the curve (AUC) for each pig profile. A Kruskal-Wallis test followed by the Wilcoxon pairwise test was then used to look for significant differences between groups. Differences in viral loads in sera for contact pigs were analyzed only at viremia peak for all animals with a Kruskal-Wallis test and a Wilcoxon pairwise test. Results with *p*-values ≤ 0.05 were considered statistically significant.

Longitudinal data from PRRSV genome detection in sera of inoculated and contact animals from the three different groups (Porci, Uni, and Rec) were used to estimate transmission parameters by a Bayesian inference, as previously described [31]. Three parameters were determined: the latency (representing the time lapse when an infected pig becomes infectious), the duration of viral shedding, both expressed in days, and the daily transmission rate or number of infected pigs by an infectious pig per day.

## 3. Results

### 3.1. Clinical Data

The vaccine and the recombinant strains did not induce significant clinical signs in comparison with the control group, with the exception of some limited temperature rises as described in the SPC for the vaccine. One inoculated pig from the Rec group was euthenazied before the end of experiment at 31 dpi independently of PRRSV infection (ethical euthanasia due to rectal prolapse).

### 3.2. Virological Parameters in Inoculated Pigs

#### 3.2.1. Viremia

**Figure 2:**
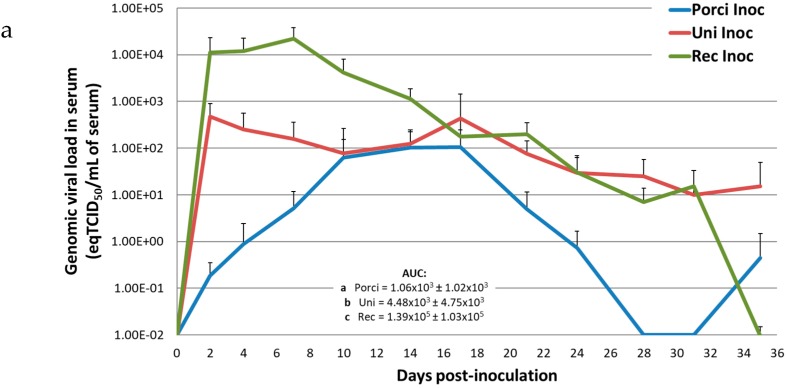

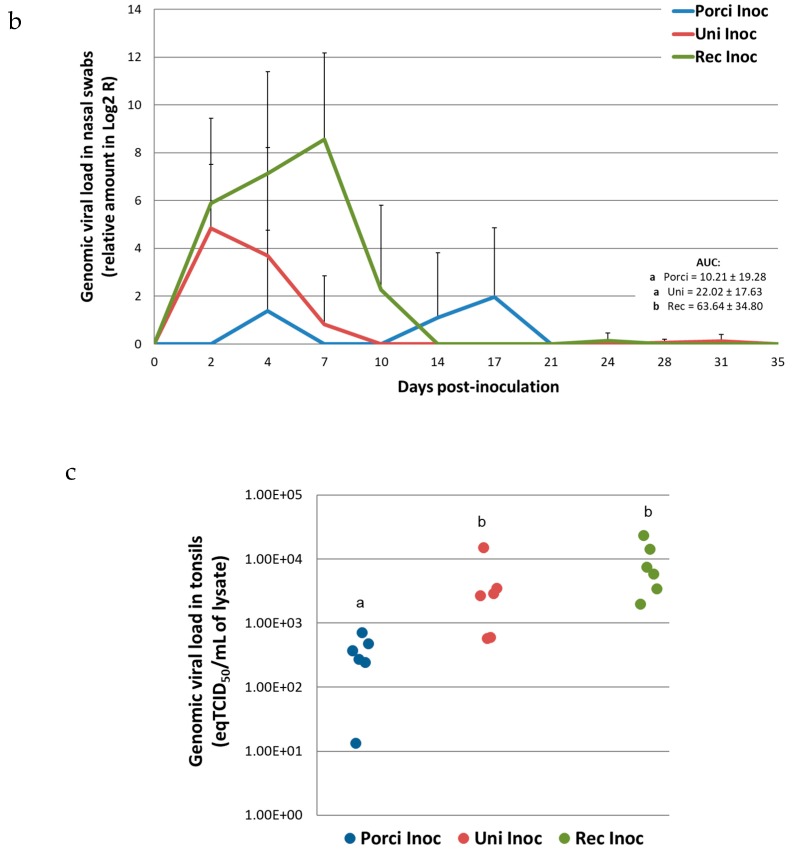
Genomic viral loads in serum, nasal swabs, and tonsils from inoculated pigs. (**a**) Evolution of the mean genomic viral loads in inoculated pigs from the Porci, Uni, and Rec groups in serum (equivalent TCID_50_/mL of serum) after inoculation (day 0). AUC: Area Under the Curve. Different letters (a, b, c) indicate that the groups are significantly different from each other with *p* ≤ 0.05. (**b**) Evolution of the mean genomic viral loads in inoculated pigs from Porci, Uni, and Rec groups in nasal swab supernatants (relative amount expressed in log2 R) after inoculation (day 0). AUC: Area Under the Curve. Different letters (a, b) indicate that the groups are significantly different from each other with *p* ≤ 0.05. (**c**) Post-mortem analysis of individual genomic viral loads in tonsils (equivalent TCID_50_/mL of tissue lysate) at 36–39 dpi in inoculated pigs from Porci, Uni, and Rec groups. Different letters (a, b) indicate that the groups are significantly different from each other with *p* ≤ 0.05.

Inoculated pigs from the different groups showed different profiles of viremia (Figure 2a). Pigs in the Uni and Rec groups quickly reached viremia peaks at 2 and 7 dpi respectively, whereas pigs in the Porci group reached a viremia plateau between 10 and 17 dpi. Then, animals in the Porci group became PRRSV negative in serum at 28 dpi. The genomic viral load was undetectable for the Rec group at 35 dpi, whereas pigs in the Uni group were still PRRSV positive at the same time. At peak, mean viremia for the Porci and Uni groups were 10^2.03^ and 10^2.67^ eqTCID_50_/mL of serum respectively, whereas in the Rec group, viral load reached 10^4.34^ eqTCID_50_/mL of serum. The mean viremia AUCs of the inoculated pigs from the three groups were all significantly different from each other (*p* ≤ 0.05) with the highest AUC for the Rec group and the lowest for the Porci group. Data obtained from the inoculated pig in the Rec group euthanized at 31 dpi were included in the quantification from 0 to 31 dpi. At 35 dpi, average genomic viral load in serum was calculated with five pigs in the Rec group. Control pigs did not show any PRRSV viremia during the experiment.

#### 3.2.2. Nasal Viral Shedding

The PRRSV genome was detected in the nasal swab supernatants of only 3 of 6 pigs in the Porci group and in 6 of 6 animals in both the Uni and Rec groups for respectively 10, 8 and 12 days. The highest viral load in nasal swab supernatants was observed in the Rec group with a relative amount (log2 R) of 8.6 at 7 dpi in comparison with the Uni (log2 R = 4.8 at 2 dpi) and Porci (log2 R = 2.0 at 17 dpi) groups. Viral nasal shedding for the Rec group was thus 2 to 4 fold higher than for Uni and Porci groups, respectively at peak. Furthermore, the mean AUC values for nasal viral loads were significantly different between the Rec and Porci groups, and between the Rec and Uni groups, with the highest value for the Rec group (*p* ≤ 0.05). No significant difference was shown between the Porci and Uni groups (*p* > 0.05) (Figure 2b). Data obtained from the inoculated pig in the Rec group euthanized at 31 dpi were used with the same methodology as for quantification of PRRSV viremia. Control animals stayed PRRSV negative in nasal swabs until the end of experiment.

#### 3.2.3. Viral Load in Tonsils

The average genomic viral load in the tonsils of inoculated pigs from the Rec group (10^3.97^ eqTCID_50_/mL of tissue lysate) was significantly higher than in the Porci group (10^2.54^ eqTCID_50_/mL, *p* = 0.0065), but not than the Uni group (10^3.63^ eqTCID_50_/mL, *p* = 0.1320) (Figure 2c). Concerning the inoculated pig from the Rec group euthanized before the end of experiment, quantification of individual genomic viral load in the tonsils was made using the tissue sample from 31 dpi collected during necropsy. No PRRSV genome was detected in the tonsils of control pigs.

### 3.3. Virological Parameters in Contact Pigs

**Figure 3:**
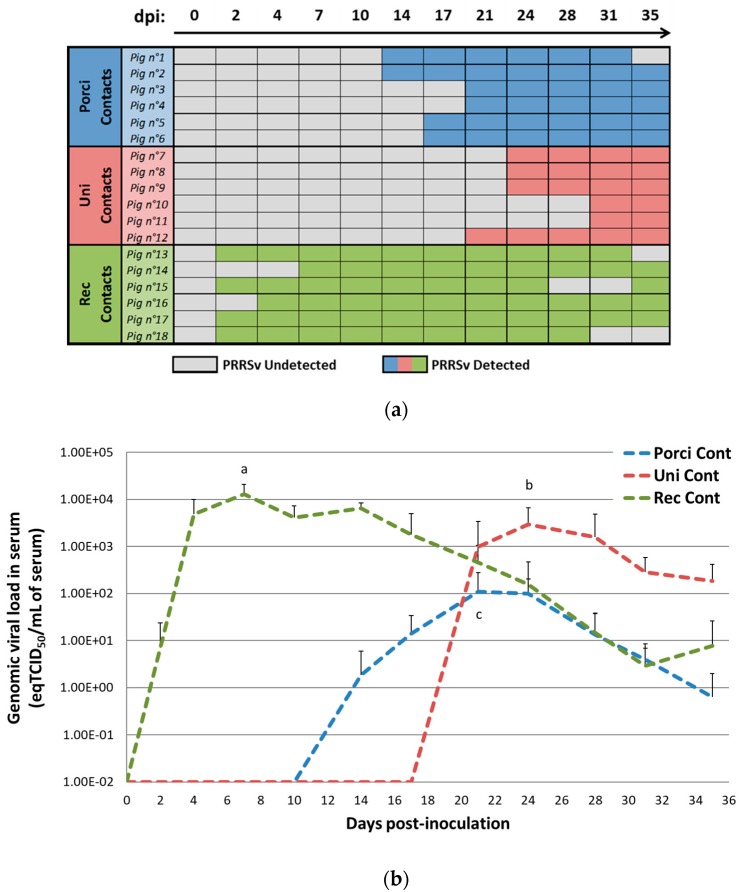
Porcine Reproductive and Respiratory Syndrome Virus (PRRSV) detection and genomic viral load in serum from contact pigs. (**a**) Detection of genomic viral loads in the serum of contact animals from the Porci, Uni, and Rec groups: individual detection data. (**b**) Evolution of the mean viremia in contact pigs after inoculation (day 0) (equivalent TCID_50_/mL of serum). Different letters (a, b, c) indicate that the groups are significantly different from each other at viremia peak with *p* ≤ 0.05.

In contact pigs, the recombinant strain was detected in 4 of 6 animals at 2 dpi (1 day after contact with the inoculated pigs) (Figure 3a). In this group, all the contact pigs were viremic at 7 dpi. In contrast, in the Porci group, the first viremic contact pig was detected at 14 dpi (13 days of contact) and all the animals were viremic at 21 dpi. Viral transmission in the Uni group was even slower with the first viremic contact pig at 21 dpi (20 days of contact) and all the pigs were viremic at 31 dpi only.

Contact pigs showed the same viremia profile, shifted in time, as inoculated animals (Figure 3b). The viremia peaks of contact animals in the Porci and Uni groups were respectively reached at 21 dpi (with 10^2.04^ eqTCID_50_/mL) and 24 dpi (with 10^3.47^ eqTCID_50_/mL), whereas maximum mean viremia was 10^4.12^ eqTCID_50_/mL at 7 dpi in the Rec group. Similarly to inoculated animals, the viremia peak of contact pigs from Rec group was about 4 to 120 fold higher than contact pigs from the Uni and Porci groups respectively. The magnitude of the viremia peak for contact animals was significantly different between the 3 groups (*p* ≤ 0.05).

### 3.4. Transmission Parameters

For the Porci and Rec groups, a similar latency was estimated: 0.5 day (0.3; 1.0), whereas pigs from the Uni group became infectious 1 day (0.6; 2.0) on average after infection (Table 1). The duration of viral shedding and daily transmission rate were significantly higher in the Rec group than in vaccine groups. Animals inoculated with the recombinant strain shed PRRSV for 34.7 days (30.6; 39.3) on average, 4 days more than pigs from the Uni group (30.1 days (26.2; 34.7)), and 11 days more than Porci group (23.3 days (20.6; 26.3)). Furthermore, the daily transmission rate in the Rec group was 0.57 (0.23; 1.23), five-fold higher than the Porci group with 0.11 (0.05; 0.22) and seven-fold higher than the Uni group with 0.08 (0.03; 0.16).

### 3.5. Genomic Evolution of the Recombinant Strain Persisting on the Farm

**Figure 4:**
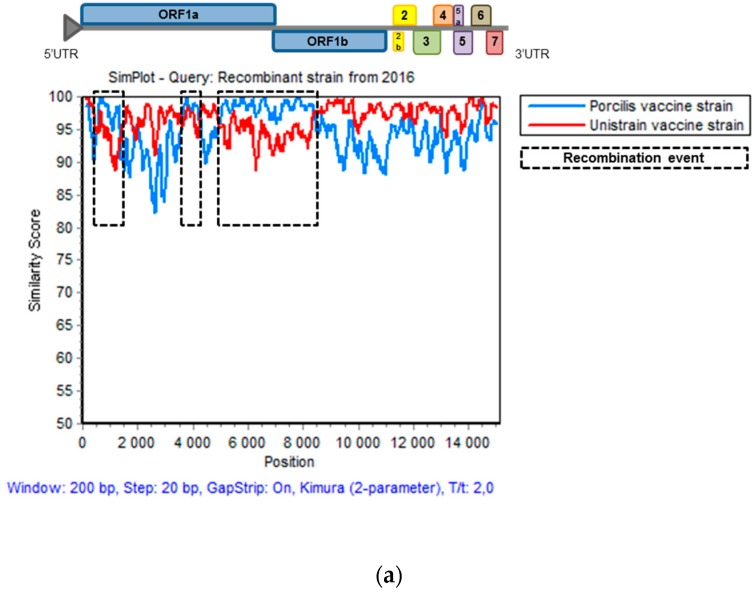

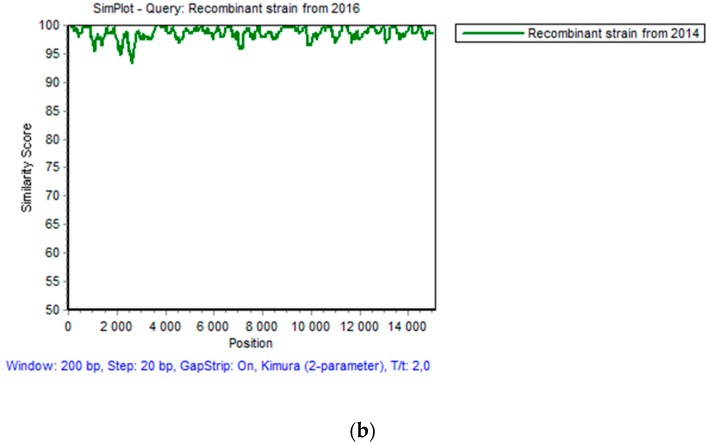
Full-genome similarities between the recombinant strain from 2016 and the vaccine strains or the recombinant strain from 2014. (**a**) Comparison of full-genome similarities with the recombinant strain isolated in 2016 and the Porcilis^®^ PRRS and Unistrain^®^ PRRS parental vaccine strains. (**b**) Comparison of full-genome similarities between the recombinant strains isolated in 2014 and 2016. Plots of similarity were generated with the Simplot program using the recombinant strain isolated in 2016 as the genome reference for both graphs.

Recombination analyses confirmed the PRRSV strain isolated in 2016 in the field was still the recombinant strain with a Unistrain^®^ PRRS vaccine strain backbone and three recombination events with Porcilis^®^ PRRS vaccine strain located in exactly the same areas as those described from the strain isolated in 2014 (Figure 4a) (Table 2). In fact, the three recombination breakpoints were all located in ORF1, including the first two events entirely located in ORF1a, and mostly in ORF1a for the 3rd breakpoint. Four chimeric non-structural proteins (NSPs) resulted from these recombinations (NSP1, NSP2, NSP3, and NSP9). The mutation rate obtained in these proteins was similar to those observed in other NSPs derived from a single parental strain as NSP10, NSP11, or NSP12 (Supplementary Table S1). Genomic comparison between both PRRSV field strains from 2014 and 2016 exhibited 98.6% identity due to 214 mutations across the whole genome and leading to the modification of 173 amino acids (Figure 4b). Comparison between the full-genome sequences of the two MLV1 strains and sequence of the field PRRSV strain isolated in 2016 showed percent identities of 96.6% with Unistrain^®^ PRRS and 94.9% with Porcilis^®^ PRRS vaccine strains whereas for the recombinant strain from 2014, percent identities were respectively 97.5% and 95.8% with Unistrain^®^ PRRS and Porcilis^®^ PRRS vaccine strains.

## 4. Discussion

Recombination is an important genetic mechanism contributing to the evolution of PRRSV and leading to emergence of novel strains, potentially exhibiting increased virulence [14].

In the current study, different parameters such as clinical signs, viremia, nasal excretion, and transmission were measured and compared between a recombinant PRRSV strain isolated from the field and both parental MLV1 strains. Even though no significant clinical signs were observed in our SPF pigs, significant differences in viral parameters were observed.

Animals infected with the recombinant strain showed a viremia level 10- to 100-fold higher in comparison with Unistrain^®^ PRRS or Porcilis^®^ PRRS vaccine strains, both in inoculated and contact pigs. Of note, the viremia profile in the Rec group was similar to that in the Uni group, i.e., exhibiting an early viral detection, a quickly reached viremia steady state, and high and persistent viral loads. This profile might be linked to the genetic origin of the recombinant strain with 2/3 of its genome originating from the Unistrain^®^ vaccine (major parent) and 1/3 originating from Porcilis^®^ vaccine (minor parent) [28]. From this point of view, the recombinant strain seems to have overpassed the replication capacities of its major parental vaccine strain in order to be more adapted to its natural host.

To explain the difference in the dynamics of viremia between the recombinant strain and the parental vaccine strains, we cannot exclude an effect of the inoculum dose. Indeed even if the three strains had equivalent titer in MARC-145 cells, the recombinant strain displayed a higher titer in PAMs compared to MARC-145 (10^4.2^ TCID_50_/mL in MARC-145 and 10^6^ TCID_50_/mL in PAMs). This could have resulted in the inoculation of a higher amount of infectious particles for the recombinant strain (compared to the vaccine strains) during the experiment. Nevertheless, even if a higher inoculum dose had been used for the recombinant strain, this should not have substantially impacted on the results of the study. Indeed previous studies have shown that for PRRSV, the viremia level is mostly independent to the inoculum dose. Loving et al., [32], demonstrated that initial PRRSV dose did not correlate with PRRSV viremia kinetics during the acute stage of infection when using a low (10^2^ CCID_50_) or high (10^6^ CCID_50_) dose of PRRSV inoculum. These results are supported by those of Haiwick et al. [33], who showed that, for a challenge dose of 10^3^ or 10^4^ TCID_50_/mL, pigs had similar post-challenge viremia profiles. Nevertheless, in groups exposed to lower inoculum doses, less than 10^1.5^ and 10^2^ TCID_50_/mL, viremia dynamics were altered with a delayed viremia peak.

The limited effect of the inoculum dose is also sustained by the results from the contact pigs of our study. Indeed contact pigs from the Rec group had the same level of viremia as Rec inoculated pigs while these contact pigs probably get infected by the nasal route with a lower dose of PRRSV than those received by inoculated pigs. Altogether these results support the hypothesis that the viremia level may be strongly linked to the fitness of the PRRSV strain but not to the inoculum dose.

The increased level of viral particles excreted via the respiratory tract is consistent with the observed highest viremia level of the recombinant strain compared with the other tested strains. This is also in line with the higher PRRSV viral load detected in tonsils from pigs in the Rec and Uni groups compared to the Porci group. Thus, the enhanced nasal excretion capacities of the recombinant strain in inoculated pigs can explain the stronger transmission of the recombinant PRRSV strain to contact pigs in the early stages of infection, compared to the vaccine strains. Using the same methodology, we previously determined the transmission parameters for a PRRSV field strain from France [31]. Interestingly, the transmission parameters of the recombinant strain were very close to those determined for this PRRSV-1 wild-type strain. Regarding the transmission of Unistrain^®^ PRRS and Porcilis^®^ PRRS vaccine strains to contact pigs, we found some unexpected results. Indeed, while viremia and nasal shedding were lower for the Porci group compared to the Uni group, contact pigs from the Porci group became infected earlier. Our results are therefore not consistent with those of Martinez-Lobo et al., [34], who found faster transmission of the Unistrain^®^ PRRS vaccine strain compared to Porcilis^®^ PRRS vaccine strain. To explain this difference in terms of viral transmission between both studies, it has been suggested that in vivo replication of attenuated vaccine strains may lead to mutant selection [35]. In our study, during the adaptation process of vaccine strains in the swine host, mutations might have conferred increased transmission capacities to Porcilis^®^ PRRS but not to Unistrain^®^ PRRS.

High transmission capacities might confer long-term persistence to the recombinant strain in the pig herd. Indeed, two years after the first isolation of the recombinant strain, we could confirm that the recombinant PRRSV strain was still circulating on the farm. The 2016 recombinant strain sequence, with 1.4% genetic divergence (due to additional mutations over the years) compared to the recombinant strain from 2014, is in line with previous data (from field experience and experimental infections) that described a mutation rate of about 0.5% to 1% per year [36,37].

Even though three recombination events were clearly identified in the field strain under study, recombination may not be the only or main reason resulting in increased transmission characteristics. Indeed, the recombinant strain was isolated from the field in December 2014 more than one year after two successive vaccinations in a limited period of time, which are most probably the source of the recombination events between the two MLV1 vaccines. The recombinant strain might have increased its replication capacities thanks to selection of more adapted virus variants following multiple pig passages in the farm. This phenomenon has already been described for MLV2 strains [38]. Mengeling et al. [39] suggest that vaccine strains could evolve during in vivo passages, enabling them to replicate more efficiently in their natural target cells. They also found that genetic reversion to virulence is a gradual process, occurring with a prolonged passage time of the strain in the field. Given our results and data from the literature, one of the major assumptions is that the adaptation process of attenuated strains after multiple passages in the pig might have changed the tropism of the virus and increased its replicative capacity. In our laboratory (data not shown), Porcilis^®^ PRRS and Unistrain^®^ PRRS vaccine strains were not able to replicate in PAMs, suggesting that they might have lost their ability to replicate in their natural target cells during the attenuation process in cell lines such as MARC-145. However, the recombinant field strain from 2014 replicated both in PAMs and MARC-145 cells, while the recombinant strain isolated in 2016 could only replicate in PAMs and lost its ability to multiply in MARC-145 cells. In fact, the replication capacities of this PRRSV strain of vaccine origin seem to have evolved after multiple in vivo passages, recovering the ability to replicate in vitro in PAMs, the natural target cells.

In this study, we characterized in vivo a field recombinant PRRSV-1 strain derived from two MLV1 strains. Although no significant clinical signs were observed, this recombinant strain demonstrated increased excretion and transmission capacities compared to parental vaccine strains. The viral fitness of this recombinant strain might reveal its safety level since propagation and persistence in the field might increase the risk of reversion to virulence due to genetic evolution over several years. Because vaccine safety in the field is an issue of particular concern, measures should be implemented to avoid any recombination phenomenon of PRRSV vaccine strains under field conditions, i.e., a more careful use of MLVs for PRRSV prevention and control.

## Figures and Tables

**Table 1:** Estimation of transmission parameters in the Porci, Uni, and Rec groups.

TRANSMISSION PARAMETERS	Porci	Uni	Rec
**Duration of viral shedding** **(days)**	23.3	30.1	34.7
	(20.6; 26.3) *	(26.2; 34.7)	(30.6; 39.3)
**Duration of latency** **(days)**	0.5	1.0	0.5
	(0.3; 0.9)	(0.6; 2.0)	(0.3; 1.0)
**Daily transmission rate** **(number of infected pigs by an infectious pig per day)**	0.11	0.08	0.57
	(0.05; 0.22)	(0.03; 0.16)	(0.23; 1.23)

* 95% credibility interval.

**Table 2:** Comparison of the three recombination events identified with RDP4 software for PRRSV recombinant strains isolated on the farm in 2014 and 2016.

		PRRS-FR-2014-56-11-1 Recombinant Strain Isolated in 2014	PRRS-FR-2016-56-11-1 Recombinant Strain Isolated in 2016
**Event No. 1**	Segment length and position	500–1370 nt	510–1408 nt
	% similarity with Porcilis	99.5%	98.0%
	*p* value RDP	3.372.10^−34^	9.727.10^−25^
**Event No. 2**	Segment length and position	3646–4272 nt	3646–4272 nt
	% similarity with Porcilis	99.5%	98.7%
	*p* value RDP	5.790.10^−17^	9.431.10^−14^
**Event No. 3**	Segment length and position	4972–8430 nt	4994–8376 nt
	% similarity with Porcilis	99.5%	98.4%
	*p* value RDP	3.894.10^−63^	6.115.10^−47^

## Supplementary Materials

The following are available online at https://www.mdpi.com/1999-4915/11/3/296/s1, Table S1: Mutation rate in non-structural proteins (NSPs) encoded by the ORF1 in the recombinant strains isolated in 2014 (Rec 2014) or 2016 (Rec 2016) compared with the parental vaccine strains.
