# Supplementary material for: A Field Recombinant Strain Derived from Two Type 1 Porcine Reproductive and Respiratory Syndrome Virus (PRRSV-1) Modified Live Vaccines Shows Increased Viremia and Transmission in SPF Pigs
Source: Viruses. 2019 Mar 23;11(3):296. doi: 10.3390/v11030296 (PMC6466261; doi:10.3390/v11030296)
Supplement: Supplementary file 1 [file viruses-11-00296-s001.pdf]

		NSP1	NSP2	NSP3	NSP4	NSP5	NSP6	NSP7a	NSP7b	NSP8	NSP9	NSP10	NSP11	NSP12
**Rec 2014**	Parental strain													
	Mutation rate (%)	**0.52**	**1.39**	**1.16**	**1.15**	**0.39**	**0.00**	**0.67**	**0.56**	**0.00**	**0.49**	**0.83**	**1.04**	**1.32**
**Rec 2016**	Parental strain													
	Mutation rate (%)	**2.01**	**2.75**	**2.17**	**1.48**	**1.76**	**0.00**	**1.79**	**3.06**	**0.74**	**1.27**	**2.34**	**2.38**	**2.19**

Unistrain vaccine strain

Porcilis vaccine strain

Supplementary Table S1:

Mutation rate in non-structural proteins (NSPs) encoded by the ORF1 in the recombinant strains isolated in 2014 (Rec 2014) or 2016 (Rec 2016) compared with the parental vaccine strains.
The recombination breakpoints occurred in NSP1, NSP2, NSP3 and NSP9 for both strains. The mutation rate was calculated from nucleotide variation encountered in nucleotide sequence of the gene encoding each protein compared to those of the corresponding parental strain. For chimeric proteins with two parental strains, the comparison was made with the corresponding parental strain according the area.
